# The Value of Neuraminidase Inhibitors for the Prevention and Treatment of Seasonal Influenza: A Systematic Review of Systematic Reviews

**DOI:** 10.1371/journal.pone.0060348

**Published:** 2013-04-02

**Authors:** Barbara Michiels, Karolien Van Puyenbroeck, Veronique Verhoeven, Etienne Vermeire, Samuel Coenen

**Affiliations:** 1 Department of Primary and Interdisciplinary Care Antwerp, Centre for General Practice, Faculty of Medicine and Health Sciences, University of Antwerp, Antwerp, Belgium; 2 Center for Research and Innovation in Care, Faculty of Medicine and Health Sciences, University of Antwerp, Antwerp, Belgium; 3 Laboratory of Medical Microbiology, Vaccine and Infectious Disease Institute (VAXINFECTIO), Faculty of Medicine and Health Sciences, University of Antwerp, Antwerp, Belgium; Cochrane Acute Respiratory Infections Group, Italy

## Abstract

Controversy has arisen regarding the effectiveness of neuraminidase inhibitors (NIs), especially against influenza-related complications. A literature search was performed to critically assess the evidence collected by the available systematic reviews (SRs) regarding the benefits and disadvantages of NIs (oseltamivir, zanamivir) compared to placebos in healthy and at-risk individuals of all ages for prophylaxis and treatment of seasonal influenza. A SR was done using the Cochrane Database of Systematic Reviews, Health Technology Assessment Database, Database of Abstracts of Reviews of Effects, and Medline (January 2006–July 2012). Two reviewers selected SRs based on randomized clinical trials, which were restricted to intention-to-treat results, and they assessed review (AMSTAR) and study quality indicators (GRADE). The SRs included (N = 9) were of high quality. The efficacy of NIs in prophylaxis ranged from 64% (16–85) to 92% (37–99); the absolute risk reduction ranged from 1.2% to 12.1% (GRADE moderate to low). Clinically relevant treatment benefits of NIs were small in healthy adults and children suffering from influenza-like illness (GRADE high to moderate). Oseltamivir reduced antibiotic usage in healthy adults according to one SR, but this was not confirmed by other reviews (GRADE low). Zanamivir showed a preventive effect on antibiotic usage in children (95% (77–99);GRADE moderate) and on the occurrence of bronchitis in at-risk individuals (59% (30–76);GRADE moderate). No evidence was available on the treatment benefits of NIs in elderly and at-risk groups and their effects on hospitalization and mortality. In oseltamivir trials, nausea, vomiting and diarrhea were significant side-effects. For zanamivir trials, no adverse effects have been reported. The combination of diagnostic uncertainty, the risk for virus strain resistance, possible side effects and financial cost outweigh the small benefits of oseltamivir or zanamivir for the prophylaxis and treatment of healthy individuals. No relevant benefits of these NIs on complications in at-risk individuals have been established.

## Introduction

In non-high-risk individuals, seasonal influenza is a self-limiting disease. Some people, such as the elderly, young children and people with concomitant morbidities, are at a higher risk for developing serious flu complications. Influenza vaccination is the best prevention method and first choice of physicians for prophylaxis [1]. Sometimes, vaccination is not available, when the vaccine is not tolerated or a mismatch between the vaccine strain and the circulating strain occurs, such as during emerging pandemics. Even vaccination is not 100% efficacious. Efficacy reaches only 40% in the elderly and there is limited good-quality evidence of the vaccine effectiveness on complications, such as pneumonia, hospitalization and influenza specific and overall mortality [2], [3], [4], [5]. Specific antiviral agents against influenza could be useful [1] for the treatment of or pre−/post-exposure prophylaxis for seasonal or pandemic influenza. The alleviation of symptoms, the reduction of antibiotic usage and the reduction of influenza-related complications such as bronchitis, otitis media, pneumonia, hospitalization and mortality are clinically relevant targets of their effect.

Among the currently available neuraminidase inhibitors (NIs), oseltamivir and zanamivir are the most widely used and tested. In Europe, a striking variation in the use of NIs is observed among different countries [6]. Viral neuraminidase enzyme activity is essential for the release of recently formed virus particles from infected cells and is thus required for the further spread of an infectious influenza virus in the body [1]. Compared with the M2 proton channel inhibitors (amantadine and rimantadine), which currently are not recommended for the prevention or treatment of seasonal influenza, the NIs are also effective against influenza B viruses, although to a lesser extent than against influenza A [7]. Zanamivir is only available for inhalation in adults and children older than five years (because the systemic absorption is limited). Oseltamivir can be taken orally (tablets or suspension) by adults and children older than one year [1]. The effect size of the NIs is inversely correlated with the time-gap between the onset of the symptoms and the start of the medication intake [8].

Recently, controversy has arisen regarding the effect of NIs against influenza-related complications [9], [10]. In several publications [9], [11], Jefferson et al. explained the difficulties that they encountered in retrieving the full reports of unpublished trials from Roche, especially those included in the review from Kaiser et al. [12], which raised a concern of reliability. As a result, the conclusions of the updated Cochrane review were changed to reflect the gap in the knowledge caused by excluding unpublished material [10].

To help clinicians and policymakers make sense of these controversies, the focus of this review was to see how the different systematic reviews (SRs) dealt with these evidence issues and to determine how these SRs represented the existing evidence. Concurrently, we aimed to synthesize the current evidence to enable clinicians to derive a management strategy.

Therefore, an extensive literature search was performed to summarize and critically evaluate the evidence collected by the existing SRs regarding the benefits and disadvantages of the use of NIs (oseltamivir, zanamivir) compared to placebos in healthy and at-risk individuals of all ages for the prophylaxis and treatment of seasonal influenza.

## Methods

### Search Strategy

#### Inclusion and exclusion criteria

Only SRs mainly based on randomized clinical trials (RCTs) that discussed the use of NIs (oseltamivir and zanamivir) for the prophylaxis and treatment of seasonal influenza and that evaluated NIs versus placebos in healthy adults, children, elderly and at-risk individuals were considered. No search was performed before 2006 because the most recently updated SRs were the focus of this review. SRs that included observational studies besides RCTs could be included, but only the results of the RCTs are shown. To respect randomization and to allow for extrapolation to current clinical practices, only the intention-to-treat (ITT) results are discussed. Narrative reviews and meta-analyses that did not systematically search the literature and did not critically assess the quality of the included trials were excluded. SRs published in languages other than English, French, Dutch or German were not eligible.

For the prophylaxis results, a distinction was made between seasonal prophylaxis, outbreak control and post-exposure prophylaxis, for which NIs were given up to 42 days, 14 days and 10 days, respectively. In prophylaxis for adults, no dosages other than those that were recommended are shown (oseltamivir, 75 mg orally once daily and zanamivir, 2×5 mg inhaled once daily). In children, dosages were adjusted according to their body weight.

In the treatment trials, only trials that used orally administered oseltamivir at 2×75 mg/day (according to weight in children) or the recommended dose of 2×10 mg/day inhaled zanamivir are shown.

#### Outcomes

The efficacy (against laboratory-proven influenza) of prophylaxis, the effectiveness in reducing the time to symptom alleviation and to a return to normal activity (as defined by the original trial protocol), the effectiveness against complications in treatment and the potential risks (adverse events) of the NIs versus placebos are the main outcomes measured. They are expressed as relative risk (RR), efficacy E = (1−RR)×100 or odds ratio (OR), unless stated otherwise in the SRs (e.g., random risk difference, mean or median difference). The most robust and reliable pooled results are presented. Absolute risk reduction was calculated where appropriate. No new pooling of results was performed.

#### Search details

First, the Cochrane Database of Systematic Reviews, the Health Technology Assessment Database (HTA) and the Database of Abstracts of Reviews of Effects were consulted using the keywords ‘influenza AND oseltamivir OR zanamivir OR neuraminidase (all fields)’ from 2006 to 2012. After checking the inclusion dates for the SRs retrieved, a PubMed search was conducted using the following search strategy: (“influenza, human”[MeSH Terms] OR (“influenza”[All Fields] AND “human”[All Fields]) OR “human influenza”[All Fields] OR “influenza”[All Fields]) AND (“neuraminidase”[MeSH Terms] OR “neuraminidase”[All Fields]) OR “oseltamivir”[MeSH Terms] OR “oseltamivir”[All Fields] OR “zanamivir”[MeSH Terms] OR “zanamivir”[All Fields] AND (Meta-Analysis[ptyp] OR Review[ptyp]) AND (English[lang] OR French[lang] OR German[lang] OR Dutch[lang]) AND (“2006/01/01”[PDAT] : “2012/08/01”[PDAT]).

### Study Selection and Data Extraction

BM and VPK selected the appropriate publications firstly on the basis of the title/abstract and secondly on the full text, applying the inclusion and exclusion criteria. The reasons for exclusion were recorded. Data were extracted by BM regarding the outcomes of the studies including the number of trials and the number of participants. In cases of disagreement, EV’s evaluation was used.

### Quality Appraisal

BM and KVP assessed the quality of the SRs using the AMSTAR tool [13]. In cases of disagreement, EV’s evaluation was used. The quality of the evidence for the individual outcomes was graded using the GRADE classification method [14] and presented according to the GRADE profiler 3.6© format (http://ims.cochrane.org/revman/gradepro). The risk of bias, inconsistency, indirectness and imprecision were considered by BM and KVP while reviewing all of the sources contributing to the evidence of the same outcome. The ‘risk of bias’ assessment of the RCTs that was focused on sequence generation, allocation concealment, blinding of participants and personnel, blinding of outcome assessment, selective reporting, incomplete outcome data and other biases with a possible impact on the final estimate of the outcome was considered [15]. The ‘risk of bias’ assessment was based on the quality of the assessments made by the selected SRs. In the case of incongruence, the original study was consulted and reassessed. The quality of the evidence (GRADE) was labeled as follows: high (no or only one problem), moderate (2 problems) or low (3 or more problems).

No formal protocol was published in English. Registration was not conducted.

## Results

**Figure 1:**
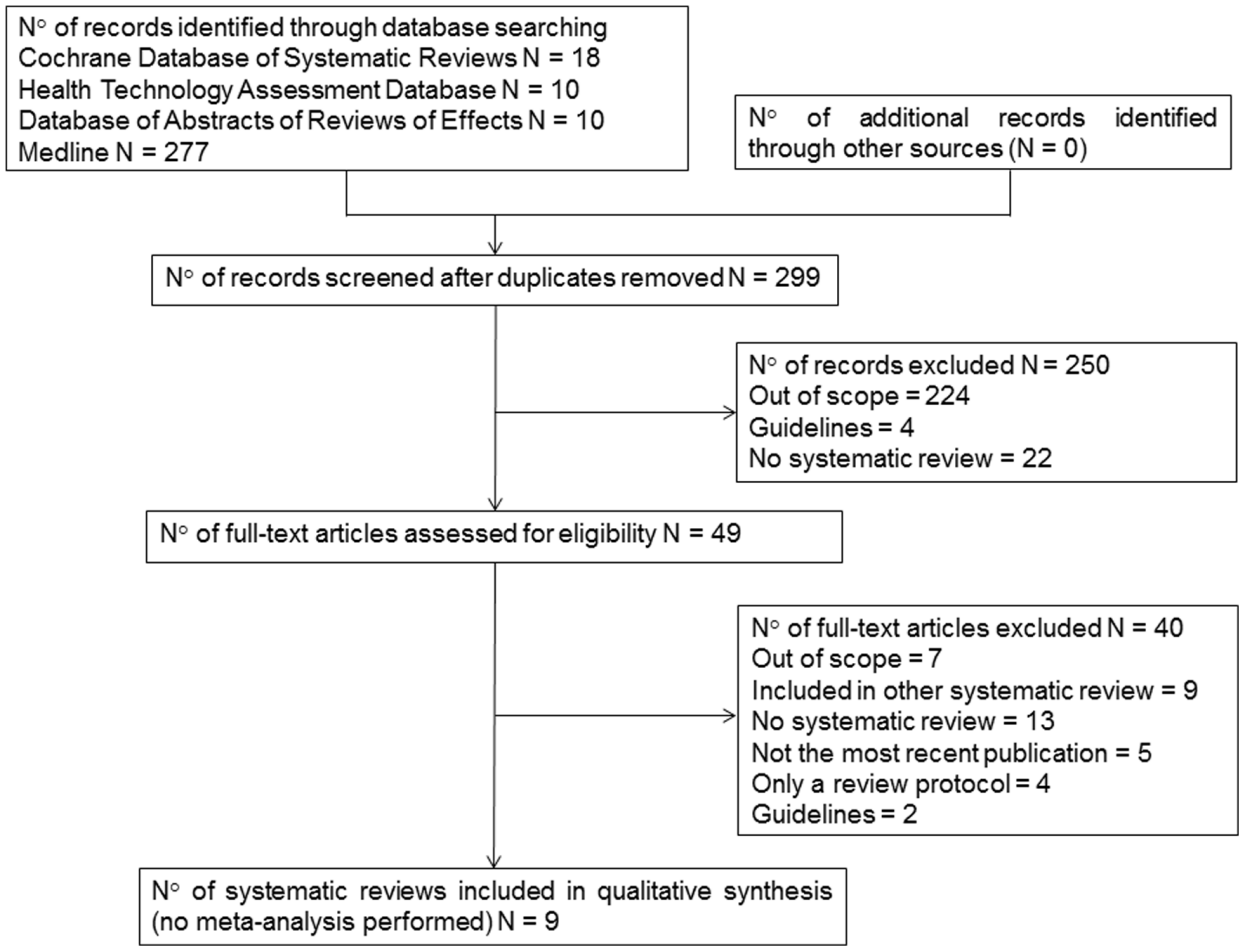
Flow of information for the search (PRISMA).

The search results are described in Figure 1. Three Cochrane reviews [10], [16], [17], two HTA clinical appraisals [8], [18] from the UK, one HTA from Canada [19] and three additional meta-analyses [20], [21], [22] were withheld. Tappenden [18], Jackson [21], Khazeni [22], Burch [8], Deonandan [19] and Falagas et al. [20] handled all ages and risk groups. Jefferson et al. [10] restricted his SR to healthy adults, children and mixed populations.

Six systematic reviews [10], [17], [18], [19], [21], [22] described the results of prophylaxis for influenza using oseltamivir and zanamivir. Khazeni et al. [22] restricted their review to the extended-duration chemoprophylaxis. Treatment results were discussed by four SRs [8], [10], [16], [17]. Falagas et al. [20] restricted his SR to the effect of NIs on influenza-related complications (Table 1).

**Table 1:** General characteristics of the included systematic reviews.

First Author/Publication year	Source	Search date up to	Intervention^a^	Prophy-laxis^b^	Treatment^b^	Effect on flu related compli-cations^b^	Adverse events^b^	Target groups	Unpublish-ed trials	Lan-guage restric-tions
Burch, 2009 [8]	HTA UK	Nov 2007	A/O/Z		N = 34	N = 19	N = 19	All ages and at-risk groups	yes	yes
Deonandan, 2007 [19]	HTA Canada	Aug 2006	O/Z	N = 11			N = 11	All ages and at-risk groups	no	no
Falagas, 2010 [20]	J Antimicrob Chemother	Sept 2009	O/Z			N = 11	N = 9	All ages and at-risk groups	no	yes
Jackson, 2011 [21]	J Infect	August 2009	A/O/Z	N = 12			N = 3	All ages and at-risk groups	no	yes
Jagannath, 2010 [16]	Cochrane SR	August 2009	O/Z		N = 0	N = 0	N = 0	Persons with cystic fibrosis	yes	no
Jefferson, 2012 [10]	Cochrane SR	April 2011	O/Z/P	N = 6	N = 19	N = 8	N = 18	Healthy adults/mixed populations/children	yes	no
Khazeni, 2009 [22]	Ann Intern Med	June 2009	O/Z	N = 6			N = 6	All ages and at-risk groups	no	no
Wang, 2012 [17]	Cochrane SR	Jan 2012	O/Z/L	N = 3	N = 6	N = 6	N = 9	Children healthy and at risk	no	no
Tappenden, 2009 [18]	HTA UK	July 2007	A/O/Z	N = 15			N = 0	All ages and at-risk groups	yes	yes

a A = amantadine; O = oseltamivir; Z = zanamivir; P = peramivir; L = laninamivir.

b N = number of trials included in SR.

The review of Jagannath et al. [16] could not retrieve any trials describing the benefits or disadvantages of NIs among persons suffering from cystic fibrosis.

The extensive HTA report of Burch et al. [8] is also summarized in The Lancet Infectious Diseases [23]. The Jackson et al. [21] SRs updated the Tappenden et al. [18] SR using the same methods and rigor.

In total, 35 reviews were excluded because of a lack of an exhaustive, systematic literature search and frequently because of a lack of critical quality appraisals for the included RCTs (Table 2). Three Cochrane reviews only showed a protocol version.

**Table 2:** List of excluded reviews with reasons.

Reference (A–Z)	Reason for exclusion
Beigel J et al. Antiviral Res. 2008 [49]	no systematic literature search, narrative review
Bettis R et al. Clin Drug Investig. 2006 [50]	no systematic literature search, narrative review
Bijl D. Int J Risk Saf Med. 2011 [51]	no systematic literature search, narrative review
Burch J et al. Lancet Infect Dis. 2009 [23]	Journal publication of Health Technology Appraisal of Burch et al. [8]
Chidiac C. Rev Prat. 2008 [52]	no systematic literature search, narrative review
Clark NM et al. Semin Respir Crit Care Med. 2011 [53]	no systematic literature search, narrative review
Dutkowski R. J Antimicrob Chemother. 2010 [54]	no systematic literature search, narrative review
Ferraris O et al. Pathol Biol (Paris). 2010 [55]	no systematic literature search, narrative review
Freemantle N et al. BMJ. 2009 [56]	no systematic literature search, narrative review; no RCTs included, evidence based on observational studies
Health Technology Assessment, 2010; HTA-32010000424 [57]	older version of Turner et al., replaced by Burch et al. [8] and Tappenden et al. [18]
Heneghan CJ. Health Technology Assessment programme, 2011, HTA-32011001126 [58]	only a protocol version, final version not available
Hernán MA et al. Clin Infect Dis. 2011 [34]	no systematic literature search, no critical quality appraisal for the included RCTs
Holzgrabe U. Pharm Unserer Zeit. 2011 [59]	no systematic literature search, narrative review
Jamieson B et al. Can Fam Physician. 2009 [60]	no systematic literature search, narrative review
Jefferson T et al. Cochrane Syst Rev. 2006 [61]	not the most recent publication of the same research group (Jefferson et al. 2012 [10])
Jefferson T et al. Lancet. 2006 [62]	journal publication of Cochrane Syst. Rev. Jefferson et al. 2006 [61]
Jefferson T et al. BMJ. 2009 [63]	evidence included in Cochrane Syst. Rev. Jefferson et al. 2010 [64]
Jefferson T et al. Cochrane Syst Rev. 2010 [64]	not the most recent publication of the same research group (Jefferson et al. 2012) (withdrawn)
Jefferson T et al. Health Technol Assess. 2010 [65]	same evidence included in the Cochrane Syst. Rev. of Jefferson et al. 2010 [64]
Jefferson T et al. Cochrane Syst Rev: 2011 [66]	only a protocol version, final version not available
Jones M et al. Expert Opin Drug Saf. 2006 [67]	Evidence included in the Cochrane syst. Rev. of Jefferson et al. 2006 [61]
Klebe G et al. Pharm Unserer Zeit. 2011 [68]	no systematic literature search, narrative review
Lee N et al. Antivir Ther. 2012 [69]	no systematic literature search, narrative review
Lynch JP et al. Semin Respir Crit Care Med. 2007 [70]	no systematic literature search, narrative review
Mallia P et al. Int J Chron Obstruct Pulmon Dis. 2007 [71]	no systematic literature search, narrative review
Matheson NJ et al. Cochrane Syst Rev. 2007 [72]	not the most recent publication of the same research group (Wang et al. [17], Jefferson et al. 2012 [10])
McCullers JA. Antivir Ther. 2011 [73]	no systematic literature search, narrative review
Moscona A. Annu Rev Med. 2008 [74]	no systematic literature search, narrative review
National Institute for Health and Clinical Excellence (NICE), 2009; HTA-32011000098 (TA-168) [40]	NICE Technology appraisal guidance based on the systematic review of Burch et al. [8]
National Institute for Health and Clinical Excellence (NICE), 2008; HTA-32011000382 (TA-67) [75]	NICE Technology appraisal guidance based on the systematic review of Tappenden P et al. [18]
Nayak JL et al. Pediatr Ann. 2009 [76]	no systematic literature search, narrative review
No author. Med Lett Drugs Ther.2006 [77]	no systematic literature search, narrative review
No author. Med Lett Drugs Ther. 2009 [78]	no systematic literature search, narrative review
No author. Med Lett Drugs Ther. 2012 [79]	no systematic literature search, narrative review
Nüesch R. Ther Umsch. 2007 [80]	no systematic literature search, narrative review
Oxford JS. Influenza Other Respi Viruses. 2007 [81]	no systematic literature search, narrative review
Preziosi P. Expert Opin Pharmacother. 2011 [82]	no systematic literature search, narrative review
Ruf BR et al. Dtsch Med Wochenschr. 2008 [83]	no systematic literature search, narrative review
Ruf BR et al. Infection Control & Hospital Epidemiology. 2009 [84]	no systematic literature search, narrative review
Salzberger B. Internist (Berl). 2006 [85]	no systematic literature search, narrative review
Schirmer P et al. Expert Opin Drug Saf. 2009 [86]	no systematic literature search, narrative review
Shun-Shin M et al. BMJ. 2009 [87]	not the most recent publication of the same research group (Wang et al. [17], Jefferson et al, 2012 [10])
Smith JR et al. Adv Ther. 2011 [88]	no systematic literature search, narrative review
Tambyah PA. Respirology. 2008 [89]	no systematic literature search, narrative review
Tappenden P et al. Health Technology Assessment, 2009; HTA-32008100360 [90]	replaced by the systematic review of Tappenden P et al. [18]
Toovey S et al. Drug Saf. 2008 [91]	no systematic literature search, narrative review
Townsend KA et al. Pharmacotherapy. 2006 [92]	no systematic literature search, narrative review
Tullu MS. J Postgrad Med. 2009 [93]	no systematic literature search, narrative review
Wang K et al. Cochrane Database Syst Rev. 2012 [94]	replaced by the systematic review of Wang et al. [17]
Wesseling G. Int J Chron Obstruct Pulmon Dis. 2007 [95]	no systematic literature search, narrative review
Whitley RJ. Expert Opin Drug Metab Toxicol. 2007 [96]	no systematic literature search, narrative review
Yang Ming et al. Cochrane Syst Rev: Protocols 2010 [97]	only a protocol version, final version not available
Yang Ming et al. Cochrane Syst Rev: Protocols 2010 [98]	only a protocol version, final version not available

### Quality Appraisal

#### Systematic reviews

In general, the SRs of Burch et al. [8], Tappenden et al. [18], Jefferson et al. [10] and Wang et al. [17] were of excellent quality according to the AMSTAR checklist [13]. Although differences were noted in their search methods, database sources, inclusion/exclusion criteria, data extraction, quality appraisals and statistical analyses, they provided an extensive description of the methods used, the quality and the general characteristics of the included and excluded trials. The latest Cochrane SR by Jefferson et al. [10] based the inclusion/exclusion criteria, the quality appraisal and the data extraction only on extensive clinical data reports, which contrasted with the other SRs that reported results based on published or short reported trials.

The SRs of Jackson [21] and Khazeni et al. [22] were also well performed, but they were only summarized in a concise publication. Although a thorough search procedure was performed in all of these SRs to unravel unpublished studies, funnel plots to assess publication bias were seldom used, and only the SR of Jefferson et al. [10] worked with a full trial list. For some outcomes, a considerable variability in the included and excluded trials exists between the different SRs. The SR of Deonandan et al. [19] was completed by one reviewer, included case-control and observational studies and did not provide useful outcome measures. The SR of Falagas et al. [20] combined different dose regimens of the NIs and only presented effectiveness results among the participants with confirmed influenza (no intention-to-treat analysis). Finally, mistakes were made in extracting the correct numbers from the original papers. The SRs from Deonandan [19] and Falagas et al. [20] did not include recent RCTs that were not yet included in other SRs. For all of these reasons, these SRs will not be discussed further (Table 3).

**Table 3:** AMSTAR quality appraisal of the included SRs.

AMSTAR questions	Burch, 2009 [8]	Deonandan, 2007 [19]	Falagas, 2010 [20]	Jackson et al, 2011 [21]	Jagannath, 2010 [16]	Jefferson, 2012 [10]	Khazeni, 2009l [22]	Wang, 2012 [17]	Tappenden, 2009 [18]
1. Was an “a priori” design provided?	yes	not specified	not specified	yes	yes	yes	no	yes	yes
2. Was there duplicate study selection and data extraction?	yes	no	yes	partially	yes	yes	yes	yes	yes
3. Was a comprehensive literature search performed?	yes	yes	yes	yes	yes	yes	yes	yes	yes
4. Was the status of publication (i.e., grey literature) used as an inclusion criterion?	yes	yes	no	yes	yes	yes	yes	yes	no
5. Was a list of studies (included and excluded) provided?	yes	partially	partially	partially	no	yes	partially	yes	yes
6. Were the characteristics of the included studies provided?	yes,	yes	yes	yes	NA	yes	yes	yes	yes
7. Was the scientific quality of the included studies assessed and documented?	yes	partially	yes	yes	NA	yes	yes	yes	yes
8. Was the scientific quality of the included studies used appropriately in formulating conclusions?	yes	no	yes	no	NA	yes	yes	yes	no
9. Were the methods used to combine the findings of studies appropriate?	yes	NA	yes	yes	yes	yes	yes	yes	yes
10. Was the likelihood of publication bias assessed?	no	no	partially, no specific tests were performed	no	NA	yes	no	partially, no specific tests were performed	no
11. Was the conflict of interest included?	yes for the reviewers, no for the included RCTs	no	unclear for the reviewers, no for the included RCTs	no	yes for the reviewers, no for the included RCTs	yes	yes for the reviewers, no for the included RCTs	yes for the reviewers, no for the included RCTs	yes for the reviewers, no for the included RCTs

SR = systematic review; NA = not applicable.

#### Original publications

Very few prophylaxis and treatment trials showed a well-reported methodology and had a minor risk of bias [8], [10]. Other prophylaxis and treatment trials were at risk of bias because of the poor description of the allocation concealment, the number of withdrawals (losses to follow-up), blinding, randomization methods and power calculations [8], although the randomization and allocation concealment of the trials were regarded as adequate in most studies by Jefferson [10]. Very few original studies published results regarding the ITT population (which indicates all of the participants with influenza-like illness (ILI)), and at least two studies were open-label [24], [25]. Complications and adverse events were poorly and possibly selectively reported or misclassified in most of the trials [10]. Adverse events similar to influenza symptoms were generally excluded from the trials [10]. Important baseline characteristics such as vaccination status and antibiotic usage were not always reported [10]. The quality of the zanamivir publications was graded better than that of the oseltamivir reports [8], [10].

### Clinical Effects

#### Prophylaxis

In healthy adults, the seasonal prophylaxis against influenza showed a significant efficacy of 76% (95% CI 42–90) for oseltamivir (GRADE moderate) corresponding with an absolute risk reduction (ARR) of 3.6% (95% CI 2.0–4.3) and 68% (95% CI 37–83) for zanamivir (GRADE moderate) – ARR = 4.1% (95% CI 2.3–5.1). For post-exposure prophylaxis, 81% (95% CI 55–92) efficacy for oseltamivir (GRADE moderate) – ARR = 7.0% (95% CI 4.8–8.0) and 79% (95% CI 67–87) for zanamivir (GRADE moderate) – ARR = 6.9% (95% CI 5.8–7.6) were shown (Table 4).

**Table 4:** Efficacy of the use of oseltamivir and zanamivir in prophylaxis against symptomatic, laboratory-confirmed influenza in healthy adults, children, elderly and at-risk individuals according to different systematic reviews (ITT – pooled results).

Outcome	NIa,b	Number of days	First author/review	Number of included studies	Number of participants	Percentage of influenza in placebo group	Estimate (95% CI)^c^	Quality GRADE^e^	References of included studies
**Healthy adults**									
Seasonal prophylaxis	O	42	*Tappenden * [*18*]	1	1039	4.8%	0.27 (0.09 to 0.83)	moderate	Hayden 1999 [99]
			*Jackson * [*21*]	1	1039	4.8%	0.24 (0.09 to 0.54)		Hayden 1999 [99]
			*Khazeni * [*22*]	2	1039/308	4.8%/13.7%	0.24 (0.10 to 0.58)/(0.24 (0.09 to 0.61)^d^		Hayden 1999 [99]/Kashiwaghi 2000 [47]
	Z	28	*Tappenden * [*18*]	2	1107/316	6.1%/3.8%	0.32 (0.17 to 0.63)/(0.49 (0.12 to 1.92)^d^	moderate	Monto 1999 [30]/GSK study 167/101
			*Jackson * [*21*]	1	1107	6.1%	0.32 (0.17 to 0.63)		Monto 1999 [30]
			*Khazeni * [*22*]	1	1107	7.8%	0.33 (0.18 to 0.59)		Monto 1999 [30]
Post-exposure prophylaxis	O	7 to 10	*Tappenden * [*18*] */Jackson * [*21*]	2	1747	8.7%	0.19 (0.08 to 0.45)	moderate	Welliver 2001 [42]/Hayden 2004 [26]
	Z	5 to 10	*Tappenden * [*18*] */Jackson * [*21*]	3	2416	8.7%	0.21 (0.13 to 0.33)	moderate	Hayden 2000 [100]/Monto 2002 [101]/Kaiser 2000 [102]
**Children**									
Post-exposure prophylaxis	O	10	*Tappenden * [*18*] */Jackson * [*21*] */* *Wang * [*17*]	1	215	18.9%	0.36 (0.15 to 0.84)	low	Hayden 2004 [26]
	O&Z	10	*Wang * [*17*]	3	863	12.8%	−0.08 (−0.12 to −0.05)	moderate	Hayden 2000 [100]/Monto 2002 [101]/WV16193
**At-risk elderly**									
Seasonal prophylaxis	O	42	*Tappenden * [*18*] */Jackson * [*21*]	1	548	4.4%	0.08 (0.01 to 0.63)	low	Peters 2001 [103]
			*Khazeni * [*22*]	1	548	4.4%	0.08 (0.01 to 0.63)		Peters 2001 [103]
	Z	28	*Tappenden * [*18*] */Jackson * [*21*]	1	1896	0.5%	0.20 (0.02 to 1.72)	moderate	LaForce 2007 [27]
**At-risk adults and adolescents (67–68% vaccinated)**									
Seasonal prophylaxis	Z	28	*Tappenden * [*18*] */Jackson * [*21*]	1	3363	1.4%	0.17 (0.07 to 0.44)	moderate	LaForce 2007 [27]
**Elderly subjects in long-term care (10% vaccinated)**									
Outbreak control	Z	14	*Tappenden * [*18*] */Jackson * [*21*]	1	489	9.2%	0.68 (0.36 to 1.27)	low	Ambrozaitis 2005 [28]

a O = Oral oseltamivir 75 mg 1×/day; dosage adjusted to weight in children.

b Z = Inhaled zanamivir 10 mg 1×/day; dosage adjusted to weight in children.

c relative risk or random risk difference (Wang).

d no pooling of results.

e GRADE quality of evidence: high; moderate; low.

ITT = intention-to-treat; NI = neuraminidase inhibitor; CI = confidence interval.

In children, only post-exposure prophylaxis studies were performed. One study with oseltamivir [26] found 64% (95% CI 16–85) efficacy (GRADE low quality) – ARR = 12.1% (95% CI 3.0–16.1). Oseltamivir and zanamivir studies combined showed an ARR of 8% (95% CI 5–12) (pooled results [17] – GRADE moderate quality).

In at-risk adults and adolescents, seasonal prophylaxis with zanamivir was determined by one study [27] to have 83% (95% CI 56–93) efficacy (GRADE moderate quality) – ARR = 4.0% (95% CI 1.6–4.4). In the at-risk elderly population of the same study, no significant efficacy was found (GRADE moderate quality). In at-risk elderly individuals, one study with oseltamivir during an influenza epidemic found 92% (95% CI 37–99) efficacy (GRADE low quality) – ARR = 1.2% (95% CI 0.8–1.3).

In the long-term care elderly, an outbreak control study [28] with zanamivir found no evidence of efficacy (GRADE low quality).

#### Treatment

Jefferson et al. [10]only published results for the effect of oseltamivir on the alleviation of symptoms and selected different studies compared to Burch et al. [8]. Pooled results showed that oseltamivir and zanamivir treatment alleviated the symptoms of influenza less than one day sooner. The time to return to normal activity could be reduced by one and half a days by oseltamivir and by less than half a day by zanamivir according to Burch et al. [8](GRADE high to moderate) (Table 5).

**Table 5:** Treatment effect of oseltamivir and zanamivir versus placebo in healthy adults, children, elderly and at-risk populations (ITT – pooled results).

Outcomes	NIa,b	First author/review	Number of included studies	Number of participants	Estimate (95%CI)^c^	Quality GRADE^e^	References of included studies
**Healthy adults**							
Time to alleviation of symptoms	O	*Burch * [*8*]	4	1410	−13.29 (−25.15 to −3.43)	moderate	Li 2003 [104]/Nicholson 2000 [105]/Treanor 2000 [106]/Roche WVI5730
		*Jefferson * [*10*]	5	3713	−21.3 (−29.59 to −12.98)		Whitley 2001 [107]/Nicholson 2000 [105]/Treanor 2000 [106]/M76001/WV15819
	Z	*Burch * [*8*]	6	2701	−0.57 (−1.07 to −0.08)	high	GSK NAIA3002/GSK NAI 3001/Hayden 1997 [108]/Mäkelä 2000 [109]/MIST 1998 [110]/Puhakka 2003 [111]
Time to return to normal activity	O	*Burch * [*8*]	3	951	−31.94 (−46.95 to −16.93)	moderate	Li 2003 [104]/Nicholson 2000 [105]/Treanor 2000 [106]
	Z	*Burch * [*8*]	4	3025	−0.37 (−0.84 to 0.09)	high	Hayden 1997 [108]/Mäkelä 2000 [109]/MIST 1998 [110]/Puhakka 2003 [111]
**Children**							
Time to alleviation of symptoms	O	*Burch * [*8*]	2	1029	−21.05 (–33.81 to −8.29)	moderate	Johnston 2005 [112]/Whitley 2001 [107]
	Z	*Burch * [*8*]	2	737	−0.94 (−1.43 to −0.46)	moderate	Hedrick 2000 [29]/GSK NAI30028
		*Wang * [*17*]	2	471/266	−0.5 (p = 0.001)/−0.5 (p = 0.04)^d^		Hedrick 2000 [29]/GSK NAI30028
Time to return to normal activity	O	*Burch * [*8*]	1	695	−30.08 (−43.35 to −16.81)	moderate	Whitley 2001 [107]
	Z	*Burch * [*8*]	1	471	−0.5 (−1.25 to 0.25)	moderate	Hedrick 2000 [29]
**Elderly**							
Time to alleviation of symptoms	O	*Burch * [*8*]	1	736	−10.00 (−45.05 to 25.05)	low	Martin 2001 [113]
	Z	*Burch * [*8*]	5	475	−1.13 (−2.90 to 0.63)	low	Boivin 2000 [114]/GSK NAI30012/Mäkelä 2000 [109]/MIST 1998 [110]/Murphy 2000 [115]
Time to return to normal activity	O	*Burch * [*8*]	3	734	−98.07 (−170.98 to −25.16)	low	Roche WVI15819/Roche WVI15876/Roche WVI15878
**At-risk individuals**							
Time to alleviation of symptoms	O	*Burch * [*8*]	2	1472	−17.84 (−36.20 to 0.52)	moderate	Martin 2001 [113]/Johnston 2005 [112]
	Z	*Burch * [*8*]	7	1252	−0.98 (−1.84 to −0.11)	moderate	Mäkelä 2000 [109]/Monto 1999 [30]/Murphy 2000 [115]/Boivin 2000 [114]/MIST 1998 [110]/Hedrick 2000 [29]/GSKNAI30012
Time to return to normal activity	O	*Burch * [*8*]	5	1134	−58.84 (−116.58 to −1.11)	low	Roche WVI15812/Roche WVI15872/Roche WVI15819/Roche WVI15876/Roche WVI15878
	Z	*Burch * [*8*]	6	613	−0.96 (−2.32 to 0.41)	low	Murphy 2000 [115]/GSK NAIB2007/Mäkelä 2000 [109]/MIST 1998 [110]/Hedrick 2000 [29]/Boivin 2000 [114]

a O = 150 mg oseltamivir daily during 5 days in adults, elderly; dosage adjusted to weight in children.

b Z = 2×10 mg inhaled zanamivir daily during 5 days in adults, elderly; dosage adjusted to weight in children.

c difference in median hours in oseltamivir trials and difference in median days in zanamivir trials.

d no pooling of results.

e GRADE quality of evidence: high; moderate; low.

ITT = intention-to-treat; NI = neuraminidase inhibitor; CI = confidence interval.

In children, treatment with oseltamivir was only described in two published studies [8]. Oseltamivir treatment alleviated symptoms less than one day sooner (GRADE moderate) and allowed a return to normal activity more than one day sooner (GRADE moderate). For treatment with zanamivir, less than one day was awarded in the alleviation of symptoms (GRADE moderate) [8]. No significant result was reached for the return to normal activity according to Burch et al. [8] (GRADE moderate).

Burch et al. [8] presented treatment results for NIs in elderly and at-risk individuals by extracting the subgroup from a mixed population out of the original studies. In the elderly, no evidence of an effect of oseltamivir (GRADE low) or zanamivir (GRADE low) on the alleviation of symptoms could be found by pooling these results. For the time to return to normal activity, only pooled results of three unpublished studies gave a significant reduction of four days for oseltamivir (GRADE low).

By pooling five unpublished study results, oseltamivir treatment showed more than a two day reduction in at-risk adults in the time to return to normal activity, but this conclusion had a low quality of evidence. No significant effect was found for the alleviation of symptoms (GRADE moderate). In the at-risk adults treated with zanamivir, a significant benefit of a one day reduction could be found for the alleviation of symptoms. No significant benefit could be shown for the reduction in the time needed to return to normal activity (GRADE low).

#### Complications

Drawing conclusions based on complications remains difficult and unreliable because of a lack of sound published data (Table 6). In healthy adults, oseltamivir treatment showed no significant effects on complications, except for a significant effect on antibiotic use by 63% (95% CI 52–71) found by Burch et al. [8], but this was not confirmed by Jefferson et al. [10]. Jefferson et al. [10] showed a significant preventive effect of zanamivir on asthma exacerbations: OR 0.54 (0.34–0.86) (pooled results – GRADE high).

**Table 6:** The rate of complications in healthy adults, children, elderly and at-risk individuals treated with oseltamivir and zanamivir versus placebo according to different systematic reviews (ITT – pooled results).

Outcome	NI a, b	First author/review	Number of included studies	Number of participants	Estimate (95% CI)^c^	Quality GRADE^e^	References of included studies
**Healthy adults**							
All types	O	*Burch * [*8*]	1	419	0.61 (0.32 to 1.13)	low	Treanor 2000 [106]
Pneumonia	O	*Burch * [*8*]	2	784	0.33 (0.03 to 3.16)	moderate	Nicholson 2000 [105]/Kashiwagi 2000 [47]
	Z	*Burch * [*8*]	1	588	1.36 (0.63 to 2.93)	moderate	Puhakka 2003 [111]
Bronchitis	O	*Burch * [*8*]	1	476	1.38 (0.43 to 4.40)	low	Nicholson 2000 [105]
	Z	*Burch * [*8*]	2	1054	1.08 (0.54 to 2.17)	moderate	Puhakka 2003 [111]/GSK NAI30011
Antibiotic usage	O	*Burch * [*8*]	2	1652	0.37 (0.29 to 0.48)	low	Deng 2004 [24]/Nicholson 2000 [105]
	Z	*Burch * [*8*]	1	276	0.68 (0.31 to 1.51)	low	Hayden 1997 [108]
Hospitalization	O	*Burch * [*8*]	3	2071	0.97 (0.33 to 2.90)	high	Deng 2004 [24]/Nicholson 2000 [105]/Treanor 2000 [106]
		*Jefferson * [*10*]	8	4696	0.95 (0.57 to 1.61)		Nicholson 2000 [105]/Treanor 2000 [106]/Whitley 2001 [107]/M76001/WV15707/WV15730/WV15812–15872/WV15819–15876–15978
	Z	*Burch * [*8*]	1	588	1.37 (0.86 to 2.17)	moderate	Puhakka 2003 [111]
GP consultation	Z	*Burch * [*8*]	1	588	1.05 (0.75 to 1.46)	moderate	Puhakka 2003 [111]
Astma exacerbation	Z	*Jefferson * [*10*]	9	5269	0.54 (0.34 to 0.86)	high	Hedrick 2000 [29]/Hayden 2000 [100]/Hayden 1997 [108]/Monto 1999 [30]/Boivin 2000 [114]/Mäkelä 2000 [109]/MIST 1998 [110]/NAIB2007
**Children**							
All types	Z	*Burch * [*8*]	2	732	0.88 (0.62 to 1.24)	moderate	GSK NAI30028/Hedrick 2000 [29]
Pneumonia	O	*Burch * [*8*]	2	1029	0.58 (0.26 to 1.28)	low	Johnston 2005 [112]/Whitley 2001 [107]
	Z	*Burch * [*8*]	1	266	0.51 (0.07 to 3.65)	low	GSK NAI30028
Bronchitis	O	*Burch * [*8*]	1	334	4.94 (0.57 to 42.74)	low	Johnston 2005 [112]
	Z	*Burch * [*8*]	2	732	1.05 (0.28 to 3.89)	moderate	GSK NAI30028/Hedrick 2000 [29]
Antibiotic usage	O	*Burch * [*8*]	1	695	0.96 (0.46 to 1.99)	low	Whitley 2001 [107]
	Z	*Burch * [*8*]	1	471	0.05 (0.01 to 0.23)	moderate	Hedrick 2000 [29]
Hospitalization	O	*Burch * [*8*]	1	695	0.20 (0.01 to 4.24)	low	Whitley 2001 [107]
	Z	*Burch * [*8*]	1	266	1.55 (0.06 to 38.36)	low	GSK NAI30028
GP consultation	Z	*Burch * [*8*]	1	266	0.85 (0.44 to 1.64)	low	GSK NAI30028
Otitis media	O	*Wang * [*17*]	1	334	−0.01 (−0.05 to 0.03)	low	Johnston 2005 [112]
		*Burch * [*8*]	1	695	0.82 (0.27 to 2.50)		Whitley 2001 [107]
	Z	*Burch * [*8*]	1	266	0.63 (0.16 to 2.40)	low	GSK NAI30028
Astma exacerbation	O	*Wang * [*17*]	1	177	−0.05 (−0.15 to 0.05)	low	Johnston 2005 [112]
**Elderly**							
All types	Z	*Burch * [*8*]	1	358	0.84 (0.54 to 1.32)	low	GSK NAI20012
Pneumonia	Z	*Burch * [*8*]	1	358	0.87 (0.17 to 4.38)	low	GSK NAI20012
Bronchitis	Z	*Burch * [*8*]	1	358	0.46 (0.20 to 1.02)	low	GSK NAI20012
Antibiotic usage	Z	*Burch * [*8*]	1	358	0.73 (0.43 to 1.24)	low	GSK NAI20012
**At-risk individuals**							
All types	Z	*Burch * [*8*]	4	575	0.73 (0.51 to 1.04)	moderate	GSK NAI30012/Boivin 2000 [114]/Mäkelä 2000 [109]/MIST 1998 [110]
Pneumonia	O	*Burch * [*8*]	1	334	0.48 (0.04 to 5.34)	low	Johnston 2005 [112]
	Z	*Burch * [*8*]	2	881	0.57 (0.15 to 2.23)	moderate	Murphy 2000 [115]/GSK NAI30012
Bronchitis	O	*Burch * [*8*]	1	334	4.94 (0.57 to 42.74)	low	Johnston 2005 [112]
	Z	*Burch * [*8*]	3	1210	0.41 (0.24 to 0.70)	moderate	Murphy 2000 [115]/GSK NAI30012/GSK NAI30020
Antibiotic usage	O	*Burch * [*8*]	1	334	0.96 (0.46 to 1.99)	low	Johnston 2005 [112]
	Z	*Burch * [*8*]	4	575	0.71 (0.47 to 1.07)	moderate	GSK NAI30012/Boivin 2000 [114]/Mäkelä 2000 [109]/MIST 1998 [110]
Hospitalization	O	*Burch * [*8*]	1	329	0.33 (0.01 to 8.14)	low	Roche NV16871
	Z	*Burch * [*8*]	1	524	0.50 (0.12 to 2.01)	moderate	Murphy 2000 [115]

a O = 150 mg oseltamivir daily during 5 days in adults, elderly; dosage adjusted to weight in children.

b Z = 2×10 mg inhaled zanamivir daily during 5 days in adults, elderly; dosage adjusted to weight in children.

c Estimate: odds ratio (Burch, Jefferson); risk difference (Wang).

d no pooling of results.

e GRADE quality of evidence: high; moderate; low.

ITT = intention-to-treat; NI = neuraminidase inhibitor; CI = confidence interval; GP = general practitioner.

In children, oseltamivir treatment did not show a significant effect on complications. In children treated with zanamivir, only one study [29] showed a reduction of 95% (95% CI 77–99) on antibiotic usage [8] (GRADE moderate).

In the elderly, no studies provided ITT results for the effect of oseltamivir on complications. No evidence of a benefit could be shown for zanamivir, but the studies on this topic are scarce (GRADE low).

In at-risk individuals, no significant effect could be found on the complications from influenza following oseltamivir treatment (GRADE low). Burch et al. [8] showed a significant effectiveness of 59% (95% CI 30–76) for zanamivir on bronchitis in at-risk individuals (GRADE moderate).

#### Adverse events

In healthy adults, nausea and vomiting were the most prominent adverse effects in the oseltamivir trials (OR 1.79 (95% CI 1.1–2.93)) (GRADE high) (Table 7).

**Table 7:** Adverse events of oseltamivir and zanamivir versus placebo in prophylaxis and treatment trials (ITT – pooled results).

Outcome	Trial	NIa,b	First author/review	Number of included studies	Number of participants	Estimate (95% CI)^c^	Quality GRADE^e^	References of included studies
**Healthy adults**								
Overall	Treatment	O	*Burch * [*8*]	4	1623	0.81 (0.59 to 1.12)	low	Deng 2004 [62]/Tan2002 [116]/Kashiwaghi 2000 [47]/Roche WV15730
		Z	*Burch * [*8*]	2	1054	1.03 (0.79 to 1.34)	moderate	Puhakka 2003 [111]/GSK NAI30011
Nausea	Prophylaxis	O	*Khazeni * [*22*]	3	1039/308/548	1.70 (1.15 to 2.50)/1.97 (0.61 to 6.42)/1.08 (0.48 to 2.40)^d^	low	Hayden 1999 [99]/Kashiwaghi 2000 [47]/Peters 2001 [103]
	Prophylaxis/Treatment	O	*Jefferson * [*10*]	9	5651	1.62 (1.17 to 2.26)	high	Treanor 2000 [106]/Nicholson 2000 [105]/Whitley 2001 [107]/Welliver 2001 [42]/M76001/WV15707/WV15730/WV15812–15872/WV15819–15876–15978
Vomiting	Prophylaxis	O	*Khazeni * [*22*]	3	1039/308/548	3.24 (1.07 to 9.88)/1.73 (0.52 to 5.78)/1.23 (0.33 to 4.54)^d^	low	Hayden 1999 [99]/Kashiwaghi 2000 [47]/Peters 2001 [103]
	Prophylaxis/Treatment	O	*Jefferson * [*10*]	9	5651	2.32 (1.62 to 3.31)	high	Treanor 2000 [106]/Nicholson 2000 [105]/Whitley 2001 [107]/Welliver 2001 [42]/M76001/WV15707/WV15730/WV15812–15872/WV15819–15876–15978
Diarrhoea	Prophylaxis	O	*Khazeni * [*22*]	2	308/548	0.69 (0.38 to 1.25)/0.81 (0.34 to 1.92)^d^	low	Kashiwaghi 2000 [47]/Peters 2001 [103]
	Treatment	O	*Jefferson * [*10*]	9	5651	0.72 (0.53 to 0.97)	high	Treanor 2000 [106]/Nicholson 2000 [105]/Whitley 2001 [107]/Welliver 2001 [42]/M76001/WV15707/WV15730/WV15812–15872/WV15819–15876–15978
Drug related	Treatment	O	*Burch * [*8*]	2	509	1.45 (0.83 to 2.53)	moderate	Li 2003 [104]/Roche WVI5730
		Z	*Burch * [*8*]	4	1406	1.11 (0.76 to 1.62)	moderate	Hayden 1997 [108]/Matsumoto 1999 [117]/Puhakka 2003 [111]/GSK NAI30011
Withdrawel from trial due to adverse events	Prophylaxis/Treatment	O	*Jefferson * [*10*]	9	5651	1.08 (0.66 to 1.76)	high	Treanor 2000 [106]/Nicholson 2000 [105]/Whitley 2001 [107]/Welliver 2001 [42]/M76001/WV15707/WV15730/WV15812–15872/WV15819–15876–15978
Serious	Prophylaxis	O	*Khazeni * [*22*]	1	308	0.33 (0.01 to 8.02)	low	Kashiwaghi 2000 [47]
		Z	*Khazeni * [*22*]	3	1107/3363/138	1.00 (0.06 to 15.98)/1.07 (0.54 to 2.11)/0.58 (0.15 to 2.35)^d^	low	Monto 1999 [30]/LaForce 2007 [27]/Webster1999 [118]
	Treatment	O	*Burch * [*8*]	3	985	0.32 (0.03 to 1.17)	moderate	Li 2003 [104]/Nicholson 2000 [105]/Roche WVI5730
		Z	*Burch * [*8*]	3	1130	1.44 (0.28 to 7.35)	moderate	Matsumoto 1999 [117]/Puhakka 2003 [111]/GSK NAI30011
**Children**								
Overall	Prophylaxis/Treatment	O&Z	*Wang * [*17*]	4	1766	−0.03 (−0.07 to 0.01)	moderate	Johnston 2005 [112]/Hedrick 2000 [29]/Whitley 2001 [107]/NAI30028
	Treatment	O	*Burch * [*8*]	1	334	0.91 (0.59 to 1.40)	low	Johnston 2005 [112]
		Z	*Burch * [*8*]	2	737	0.88 (0.62 to 1.24)	moderate	Hedrick 2000 [29]/NAI30028
Nausea	Prophylaxis/Ttreatment	O&Z	*Wang * [*17*]	4	1766	−0.01 (−0.03 to 0.00)	moderate	Johnston 2005 [112]/Hedrick 2000 [29]/Whitley 2001 [107]/NAI30028
Vomiting	Treatment	O	*Wang * [*17*]	3	1435	0.06 (0.03 to 0.10)	high	Heinonen 2010 [119]/Johnston 2005 [112]/Whitley 2001 [107]
		Z	*Wang * [*17*]	2	737	0.00 (−0.02 to 0.02)	moderate	Hedrick 2000 [29]/NAI30028
Diarrhoea	Prophylaxis/Treatment	O&Z	*Wang * [*17*]	5	2172	−0.01 (−0.03 to 0.00)	moderate	Heinonen 2010 [119]/Johnston 2005 [112]/Hedrick 2000 [29]/Whitley 2001 [107]/NAI30028
Drug related	Treatment	Z	*Burch * [*8*]	2	737	1.32 (0.59 to 2.92)	moderate	Hedrick 2000 [29]/NAI30028
Serious	Prophylaxis/Treatment	O&Z	*Wang * [*17*]	5	2172	0.0 (0.0 to 0.01)	moderate	Heinonen 2010 [119]/Johnston 2005 [112]/Hedrick 2000 [29]/Whitley 2001 [107]/NAI30028
	Treatment	O	*Burch * [*8*]	1	695	1.54 (0.25 to 9.24)	low	Whitley 2001 [107]
		Z	*Burch * [*8*]	2	737	2.29 (−0.24 to 22.09)	low	Hedrick 2000 [29]/GSK NAI30028
Withdrawel from trial due to ad-verse events	Prophylaxis/Treatment	O&Z	*Wang * [*17*]	3	1143	0.01 (−0.02 to 0.03)	moderate	Heinonen 2010 [119]/Hedrick 2000 [29]/NAI30028
**At-risk individuals**								
Overall	Treatment	O	*Burch * [*8*]	2	452	0.96 (0.63 to 1.46)	moderate	Lin 2004 [25]/Johnston 2005 [112]
		Z	*Burch * [*8*]	4	1286	1.24 (0.96 to 1.60)	high	Murphy 2000 [115]/MIST 1998 [110]/GSK NAI30020/GSK NAI30012
Drug related	Treatment	Z	*Burch * [*8*]	1	524	1.01 (0.55 to 1.85)	moderate	Murphy 2000 [115]
Serious	Treatment	Z	*Burch * [*8*]	3	1210	0.72 (0.32 to 1.62)	low	Murphy 2000 [115]/GSK NAI30020/GSK NAI30012

a O = treatmet trial 150 mg oseltamivir daily during 5 days in adults, elderly; dosage adjusted to weight in children; prophylaxis trial = treatment dosage/2.

b Z = treatmet trial 2×10 mg inhaled zanamivir daily during 5 days in adults, elderly; dosage adjusted to weight in children; prophylaxis trial = treatment dosage/2.

c odds ratio and risk difference (Wang).

d no pooling of results.

e GRADE quality of evidence: high; moderate; low.

ITT = intention-to-treat; NI = neuraminidase inhibitor; CI = confidence interval.

In healthy adults and children, no significant adverse effects were recorded in the treatment trials with zanamivir.

In children and at-risk individuals treated with oseltamivir or zanamivir, no significant overall drug-related or serious adverse effects could be found (pooled results) [8].

## Discussion

### Summary

The nine systematic reviews retrieved were of high quality, but they differed in their inclusion/exclusion criteria, in their quality assessment, in their data handling and finally in their conclusions. Many quality shortcomings about the included published and unpublished trials were reported.

In seasonal prophylaxis of laboratory-proven influenza, oseltamivir and zanamivir showed more than 50% effective in healthy adults and at-risk individuals (moderate to low quality). Post-exposure prophylaxis with both NIs proved to be more than 50% effective in healthy adults and children (moderate to low quality).

In healthy adults and children with ILI, both NIs showed a small treatment benefit of half a day and less than one day in the alleviation of symptoms (high to moderate quality). In elderly individuals with ILI, no significant reduction of illness days could be shown for both NIs (low quality). In at-risk individuals, no significant effect could be found for oseltamivir (moderate quality), while zanamivir showed a benefit of almost one day (moderate).

Zanamivir exclusively showed a preventive effect on antibiotic usage in children. In the prevention of influenza complications in the elderly, no benefit could be found for oseltamivir or zanamivir, but studies are scarce and of low quality in that area. In an at-risk population, an effect could be shown for zanamivir on the occurrence of bronchitis (moderate quality).

The different trials poorly reported adverse effects. In the prophylaxis and treatment studies among healthy adults and children, nausea and vomiting were prominent for oseltamivir. In at-risk individuals, no adverse effects were significant in the limited number of treatment trials, although one reviewer found more vomiting among children treated with oseltamivir. Zanamivir treatment showed no adverse effects.

### Results in Perspective

It is disappointing to find that the different NI trials focused on healthy adults rather than on the elderly and individuals at risk of developing serious influenza complications. Additionally, the choice of a primary outcome such as alleviation of symptoms or return to normal activity with a corresponding small benefit has limited clinical importance [8]. On the other hand, the effect on complications was only estimated as a secondary outcome, and trial results were often unpublished. This makes the evidence of this clinically relevant outcome a source for discussion. The trials were not designed or powered to give results regarding serious complications, hospitalization and mortality. The meta-analyses, performed by the pharmaceutical companies (Monto 1999 [30], Lalezari 2001 [31], Kaiser 2003 [12]), were of limited quality and partly based on unpublished material that was not submitted for peer-review. The methodological shortcomings of the Kaiser review [12] triggered the Cochrane review group [10] to rely only on clinical trial reports containing published and unpublished trial results, which were retrieved from the regulatory authorities and the pharmaceutical companies that produce oseltamivir and zanamivir (Roche and GSK) [32]. This collection of trial reports is on-going for zanamivir because no prophylaxis or treatment results were given for zanamivir by the latest Cochrane review by Jefferson et al. [10]. For oseltamivir, this review only considered the treatment effect on the alleviation of symptoms and on hospitalization. Other outcomes were not analyzed because of a high risk of bias. After the inclusion date of our review, Ebell et al. reported an independent meta-analysis about the effectiveness of oseltamivir treatment in adults including published and unpublished results. They concluded that no evidence of an effect could be found on hospitalization, pneumonia or the combined outcome of pneumonia, otitis media and sinusitis in the ITT population [33]. Additionally, the underreporting of side-effects was a second reason for the Cochrane reviewers to reconsider their conclusions [10]. Oseltamivir might provoke undesired neuro-psychiatric reactions such as hallucinations, suicidal tendencies and sudden death [10]. Interesting new hypotheses were tested and confirmed (post-protocol analysis) such as the difference in adverse event rates between the placebo groups of the oseltamivir and zanamivir trials and the lower antibody response in the oseltamivir groups with consequential bias (underreporting of confirmed influenza cases in the active treatment groups) [10]. On the request of Roche, Hernán et al. [34] reanalyzed the Kaiser review and added one new RCT without performing an independent, systematic literature search or quality appraisal of the included trials. No characteristics about the participants were provided. The reviewers tried to avoid the analytical problems that occurred in the Kaiser review and concluded that oseltamivir reduced the risk of lower respiratory tract complications requiring antibiotic treatment by 28% (95% CI 11 to 42%) [34]. The Cochrane Neuraminidase Inhibitors Review Team [10], [35] made critical comments on this re-analysis, which elicited a reply by Hernán et al. [36] and thereby illustrates the ongoing discussion.

The recent meta-analysis of Falagas et al. [20] of intermediate quality stated that NIs are generally effective in preventing influenza-related complications in healthy and at-risk persons, but data were only given for the subgroup with proven influenza infections. Data on individual complications were scarce and statistically insignificant.

Notwithstanding all of these shortcomings and the limited evidence of benefits that exist, many guidelines advise the use of NIs in people at risk for influenza-related complications, including individuals with chronic respiratory, cardiac, liver and renal disorders, diabetes and immunosuppression or for elderly living in nursing homes [37], [38], [39], [40], [41]. For prophylaxis, the first choice is influenza vaccination, but NIs could be considered in cases of non-vaccination or following a mismatch between the vaccine and circulating strains in at-risk groups according to the international guidelines [37], [38], [39], [40], [41]. Cost-effectiveness seems favorable for the use of NIs to treat influenza in at-risk populations, although cost-effectiveness studies are based on many assumptions, especially regarding the exact estimates of the risk and effect size of NIs on secondary complications and mortality [8].

An extra argument to use NIs might be the favorable effect on eliminating the transmission of the virus. Although virus production and excretion are slightly reduced in treated individuals, they are never completely blocked, and this claim by Roche [42] has never been proven [10]. The combination of other preventive measures such as influenza vaccination and non-pharmaceutical measures such as social distancing, case isolation, hand washing and the use of masks, is more appropriate and effective [43], [44].

In addition to the limited usefulness of NIs, a growing number of resistant influenza strains [45], especially those resistant to oseltamivir (up to 98% in the 2008–2009 season according to the WHO and ECDC), might make NIs unusable in the future [8].

### Limitations of This Review

This search focused exclusively on SRs dealing with the use of the NIs oseltamivir and zanamivir against seasonal influenza. Very few included SRs actually gave results for the newer NIs, such as peramivir and laninamivir. Only the SR of Wang et al. reported the study results of one trial on the treatment effect of laninamivir in children [46]. Guidelines discussed the prophylaxis and treatment of pandemic influenza based on the existing evidence on seasonal influenza and by extrapolating the same evidence. To avoid bias and stay close to the clinical and diagnostic uncertainty, only ITT studies were shown in this review. Publications in other languages than English, French, Dutch and German were excluded. However, by rerunning the search without language restrictions, we had no indication that we were missing any relevant reviews.

#### Some limitations and difficulties were met in the comparison of the different SRs

The different inclusion/exclusion criteria for trials that were used in the different reviews influenced the pooled outcomes. Wang [17], Jackson [21] and Khazeni et al. [22] did not use unpublished trial results compared to the other included SRs that did. Some trial results remained unpublished as extensively stated by Jefferson et al. [10], [32]. Tappenden [18] and Burch et al. [8] did not include trials that were published in Chinese or Japanese, which gave rise to translation problems. Jefferson et al. [10] did not make subgroups that the original researchers did not predefine, while others such as Burch et al. [8] defined subgroups consequently out of a mixed population by diminishing nominators and denominators accordingly. The methods used by Burch et al. [8] are prone to bias by eliminating randomization.

Jefferson et al. [10] pooled the data for both adults and children together, which makes separate conclusions for each population difficult. From the same editorial group (Cochrane Acute Respiratory Infections Group), the review of Wang et al. [17] on the effect of NIs among children only showed pooled results for both oseltamivir and zanamivir treatments together. Therefore, no distinct conclusions can be made for the NIs separately. In trials where more than one treatment group was compared with the placebo group, each reviewer handled the numbers differently. Jefferson [10] added the numbers of all of the different treatment groups, which made the intervention heterogeneous. Khazeni [22] also added results from two treatment groups and doubled the placebo numbers, which inflates the relevance of this study in the pooled results. It is unclear why Khazeni et al. [22] gave different event numbers for the Kashiwaghi [47] and Monto et al. [30] studies. Comparison with the originally published results and between the different reviews required some effort, especially where significant differences occurred between the reviews.

Most trials were designed and sponsored by Roche or GlaxoSmithKline, and independent studies are scarce. In addition to the differences in reporting quality, graded as moderate by most of the reviewers, the published trials showed differences in the number of participants, vaccinated participants, and participants with laboratory-proven influenza and treatment days, and the trials showed a different day for the assessment of the outcomes. They included different age categories and mixed healthy and at-risk people, rarely mentioning results for subgroups separately. The inclusion of participants was restricted to those suffering from influenza-like symptoms for less than 36 to 48 hours after the onset of illness. All of the treatment studies had high percentages of laboratory-proven influenza (up to 80%) [10] because they performed the studies only during influenza epidemics and excluded atypical cases. Therefore, any extrapolation of the results to the real clinical situation is limited. By consequence, their results in a subgroup of participants with laboratory-proven influenza (not shown) were only slightly better than the ITT results. The participants assessed the outcomes such as ‘alleviation of symptoms’ and ‘return to normal activity’ themselves, which introduced variability among the different trials. These outcomes were then represented in different ways: according to ITT or per protocol; or according to ILI or laboratory-proven influenza ( = subgroup). Complications such as pneumonia, bronchitis, sinusitis and otitis media were diagnosed in different ways, mostly without a clear definition and without measuring severity. No clear distinction was made between adverse events and complications. All of this heterogeneity is a source for different conclusions and recommendations.

### Recommendations for the Future

New RCTs need to focus on at-risk participants and measure severe influenza complications as an outcome, which must be powered accordingly. This also applies to the more recently developed NIs, peramivir and laninamivir, which were not discussed in this review. Head-to-head studies between oseltamivir and zanamivir and with the newer NIs might be valuable. Overall, the use of NIs has to be established among other prevention and treatment options for influenza.

The effect size of NIs is positively correlated with the accuracy and speed of the clinical diagnosis of influenza. Rapid point of care tests are promising for optimizing accuracy, but their place in the clinical diagnosis still has to be established [48].

In the future, a new policy should be established regarding the ownership of trial results. All of the stakeholders should acquire full access to clinical data reports and individual study results to avoid publication bias and selective reporting afterwards.

### Conclusion

In healthy adults and children, prophylaxis or treatment of ILI is not recommended, although effectiveness has been shown. The combination of diagnostic uncertainty, risk for virus strain resistance, side-effects and financial cost outweighs the small benefits. Prophylaxis of at-risk and elderly groups might be considered in individual cases when influenza vaccination did not take place, when it is not appropriate or is ineffective because of virus strain mismatch, when influenza is circulating in the community and when contact with an infected person could not be avoided by other measures. No evidence is available that shows a benefit for treatment in elderly and at-risk individuals, vaccinated or not, on relevant outcomes such as hospitalization and mortality.

## Supporting Information

Checklist S1 **PRISMA 2009 checklist.**
(DOC)
